# Measuring hospital antibiotic consumption in EU/EEA countries: comparison of different metrics, 2017 to 2021

**DOI:** 10.2807/1560-7917.ES.2024.29.46.2400317

**Published:** 2024-11-14

**Authors:** Igor Rubinić, Vivian H Leung, Liselotte Diaz Högberg, Dominique L Monnet, Vera Vlahović-Palčevski, ESAC-Net study group, ESAC-Net study group, Majda Attauabi, Jan Baltink, Ria Benko, Hege S Blix, Lucy Catteau, Catherine Dumartin, Filomena Fortinguerra, Emre U Gurpinar, Anna M Halldórsdóttir, Isavella Kyriakidou, Antonio López-Navas, Ragda Obeid, Anna Olczak-Pieńkowska, Gabriel A Popescu, Stéphanie Saleh, Ieva Saliete,, Emmi Sarvikivi, Birgitta Schweickert, Janne Sepp, Ana Silva, Ivan Stoikov, Reinhild Strauss, Maja Šubelj, Arjana Tambić Andrašević, Tomáš Tesar, Rolanda Valinteliene, Lenka Vostalova, Peter Zarb.

**Affiliations:** 1 Department of Clinical Pharmacology, Clinical Hospital Center Rijeka, Rijeka, Croatia; 2 University of Rijeka, Faculty of Medicine, Rijeka, Croatia; 3 European Centre for Disease Prevention and Control, Stockholm, Sweden; 4 The members of the ESAC-Net study group are listed under Collaborators

**Keywords:** hospital antibiotic consumption, multi-country analysis, defined daily dose (DDD), DDD per 100 bed-days, DDD per 100 discharges, DDD per 1 000 inhabitants per day, denominator, metrics

## Abstract

**Background:**

Antibiotic resistance poses a considerable public health threat, with data-driven stewardship a main prevention measure. While quantifying antibiotic consumption is a key component of antibiotic stewardship programmes, the choice of denominator for calculating this metric can impact comparative analyses and trend evaluations substantially, influencing targeted stewardship interventions.

**Aim:**

We aim to evaluate how using hospital sector-specific antibiotic consumption rate denominators at country level impacts country rankings and trends, addressing the limitations of the commonly used 'defined daily doses (DDD) per 1,000 inhabitants per day' metric.

**Methods:**

Hospital antibiotic consumption data from ESAC-Net and denominator data from Eurostat (‘inhabitants,’ ‘bed-days’ and ‘discharges’) for 2017–2021 were used to calculate hospital antibiotic consumption rates for 24 reporting European Union/ European Economic Area (EU/EEA) countries. Countries were ranked by their consumption rates and trends were analysed to assess the effects of using different denominators.

**Results:**

Country rankings and 5-year trend analyses varied depending on the denominator used. Antibiotic consumption rates were more similar when using hospital activity-based denominators ‘bed-days’ and ‘discharges’ compared with the population-based ‘inhabitants’ denominator. Differences in country rankings and trends were also seen among rates derived using ‘bed-days’ and ‘discharges’.

**Conclusion:**

The study underscores the importance of using hospital activity-based denominators such as ‘bed-days’ and ‘discharges’ when evaluating hospital antibiotic consumption. ESAC-Net’s historical reliance on only ‘DDD per 1,000 inhabitants per day’ is challenged, advocating for the use of multiple hospital activity-based denominators. Corresponding hospital activity denominators for ESAC-Net data will more effectively inform national hospital antibiotic stewardship interventions.

**Key public health message**

**What did you want to address in this study and why?**

We explored potential benefits and challenges of using different population- and activity-based denominators when measuring antibiotic consumption in the hospital sector in EU/EEA countries. Our results help to better understand data on antibiotic consumption.

**What have we learnt from this study?**

We found that using different denominators for calculation of national antibiotic consumption rates in the hospital sector has notable impacts on comparative analyses and trend evaluation. We also found interpretation of hospital sector antibiotic consumption rates at a multi-country level to be challenging, as the definition of hospitalised patients can differ across countries.

**What are the implications of your findings for public health?**

Measurement of antibiotic consumption is important for determining strategies to preserve antibiotic effectiveness, thus slowing the development of antibiotic-resistant bacteria. Despite challenges in obtaining hospital activity denominators such as ‘bed-days’ and ‘discharges’, more insights on antibiotic consumption in the hospital sector could be gained if harmonised definitions for hospitalisation could be applied in multi-country analyses.

## Introduction

Antimicrobial resistance (AMR) is identified as a major public health threat, both globally [1] and in Europe [2]. Emergence of antibiotic-resistant bacteria is an inevitable consequence of the widespread use of antibiotics, and ensuring prudent use of antibiotics is essential for effective AMR response. Quantifying antibiotic consumption is a crucial element of an AMR control programme, and the availability of standardised and reliable antibiotic consumption data is essential to monitor trends and to guide and evaluate antibiotic stewardship interventions.

Various metrics have been developed to monitor antibiotic consumption, with ‘defined daily doses (DDD) per 1,000 inhabitants per day’ being one of the most used metrics. It is widely used by both national and international antibiotic consumption surveillance programmes, including the European Surveillance of Antimicrobial Consumption Network (ESAC-Net) [3]. While the general population denominator used for this metric might appropriately represent the population under surveillance for antibiotic consumption in the community sector, it lacks equal relevance for antibiotic consumption in the hospital sector as it does not refer to the population under surveillance, i.e. hospitalised patients. Historically, ESAC-Net has used ‘DDD per 1,000 inhabitants per day’ as the metric for reporting antibiotic consumption in the hospital sector because uniformly defined hospital denominator data have not been available in a timely manner. In addition, it enables cross-sectoral comparison. Denominators that better reflect the hospital population under surveillance or hospital activity, such as the number of occupied bed-days or the number of discharges from hospitals, have been suggested as metrics for hospital antibiotic consumption. Reporting multiple metrics simultaneously for a more comprehensive assessment of hospital antibiotic consumption has also been suggested [3-11].

The significance of choosing an appropriate denominator for measuring hospital antibiotic consumption was underscored during the COVID-19 pandemic when hospital activity rapidly changed with considerable alterations in patient occupancy rates and hospital length of stay. National studies and reports showed contrasting trends in hospital antibiotic consumption during the pandemic period depending on the choice of denominator, indicating that using ‘DDD per 1,000 inhabitants per day’ as a sole metric was not sensitive enough to evaluate changes in hospital antibiotic consumption [12-14].

In this study, we evaluate the impact of using alternative hospital sector-specific metrics with national-level hospital antibiotic consumption data from ESAC-Net. Furthermore, we discuss the potential applications and challenges of using these metrics within international surveillance initiatives.

## Methods

### Data sources

To calculate hospital sector antibiotic consumption rates, data on antibiotic consumption (antibacterials for systemic use (Anatomical Therapeutic Chemical (ATC) J01 code)) in the hospital sector reported to ESAC-Net between 2017 and 2021 were retrieved from The European Surveillance System (TESSy) database on 18 January 2024. Antibiotic consumption data were collected using the ATC/DDD methodology. The numbers of DDD were calculated using the ATC Index for 2023 [15], as described in the ESAC-Net reporting protocol [16]. For countries with incomplete national coverage of hospital antibiotic consumption data during certain years (Portugal and Luxembourg), coverage percentages were used to extrapolate the national annual hospital antibiotic consumption in DDD.

The population-based denominator ‘inhabitants’, for calculating antibiotic consumption rates was obtained from the Eurostat dataset for ‘population on 1 January’ for each corresponding year [17]. The hospital activity-based denominators ‘discharges’ and ‘bed-days’ were obtained from the Eurostat dataset for ‘hospital discharges and length of stay for inpatient curative care’ [18]. Eurostat’s definition of ‘discharge’ is the formal release of a patient from a hospital, encompassing instances of finalised treatment, signing out against medical advice, transfers to another healthcare institution or death. The definition included healthy newborns during the years for these analyses; however, several countries noted exceptions to this when reporting to Eurostat [19,20]. The dataset used specifically covers ‘inpatients,’ referring to patients admitted overnight, and excludes ‘day cases’. ‘Bed-days’, also referred to as ‘(in)patient days’ or ‘occupied bed-days’, are defined as days during which a person admitted as an inpatient is confined to a bed and stays overnight in a hospital with the admission day and the discharge day counted together as one single day [19,20]. ‘Curative care’ refers to ‘healthcare services during which the principal intent is to relieve symptoms or to reduce the severity of an illness or injury, or to protect against its exacerbation or complication that could threaten life or normal function’ and excludes ’other functions of care (such as rehabilitative care, long-term care and palliative care)’ [21].

### Descriptive and statistical analysis

Calculations for hospital antibiotic consumption rates using all three denominators were performed for all European Union/ European Economic Area (EU/EEA) countries for which both the numerator (sum of DDD of antibiotics consumed in the hospital sector) and denominators (‘inhabitants per day’, ‘bed-days’ and ‘discharges’) were available.

To examine the effect of different denominators on the ranking of countries by antibiotic consumption, countries were ranked according to their 2021 hospital sector antibiotic consumption rates using each of the three denominators.

To examine the effect of different denominators on the 5-year (2017–2021) trends in hospital sector antibiotic consumption, a Mann-Kendall trend test and calculation of the compound annual growth rate (CAGR) were conducted for the 20 countries with all numerator and denominator data available for all 5 years. Microsoft Excel statistical tool version 16.43 and XLSTAT software version 2023.3.0 (Lumivero, Denver, United States) were used. A p value ≤ 0.05 for the Mann-Kendall trend test was considered statistically significant. The CAGR estimates the mean annual change of antibiotic consumption as a proportion (%) of the consumption in the year of commencement (2017).

## Results

For 2021, complete data were available from both data sources (ESAC-Net and Eurostat) for 24 of the 30 EU/EEA countries (Austria, Belgium, Bulgaria, Croatia, Czechia, Estonia, Finland, France, Hungary, Iceland, Ireland, Italy, Latvia, Lithuania, Luxembourg, the Netherlands, Norway, Poland, Portugal, Romania, Slovakia, Slovenia, Spain and Sweden).

**Figure 1:**
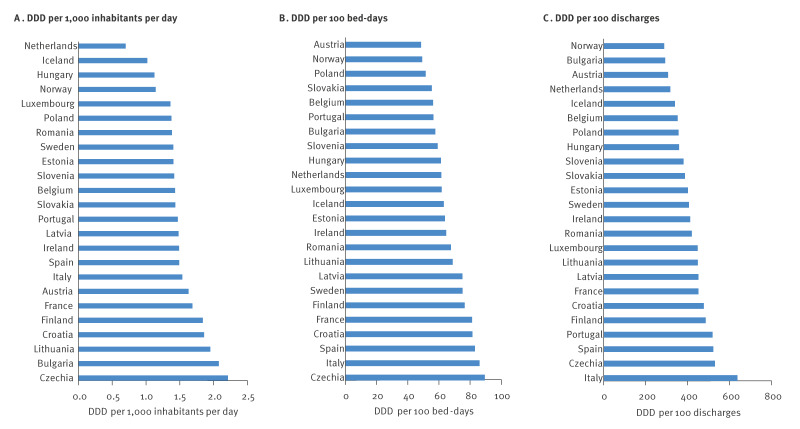
Hospital sector antibiotic consumption rates expressed and ranked according to defined daily doses per (A) 1,000 inhabitants per day, (B) 100 bed-days and (C) 100 discharges, 24 EU/EEA countries, 2021

In 2021, antibiotic consumption rate in the hospital sector varied between 0.70 and 2.21 when expressed as DDD per 1,000 inhabitants per day; between 48.5 and 89.5 when expressed as DDD per 100 bed-days; and between 288.1 and 637.7 when expressed as DDD per 100 discharges. The ranking of countries according to their antibiotic consumption rate varied greatly, depending on the metric used. The country with the lowest hospital antibiotic consumption rate was the Netherlands for the metric DDD per 1,000 inhabitants per day, Austria for DDD per 100 bed-days and Norway for DDD per 100 discharges. Italy had the highest antibiotic consumption rate expressed as DDD per 100 discharges, the second-highest expressed as DDD per 100 bed-days, but the eighth highest expressed as DDD per 1,000 inhabitants per day. Czechia had the highest antibiotic consumption rate expressed as DDD per 1,000 inhabitants per day and DDD per 100 bed-days (Figure 1).

DDD: defined daily doses; EU/EEA: European Union/ European Economic Area.

Consumption of antibacterials for systemic use were obtained using the Anatomical Therapeutic Chemical code J01 as reported to the European Surveillance of Antimicrobial Consumption Network (ESAC-Net) and retrieved from The European Surveillance System (TESSy) database.

**Figure 2:**
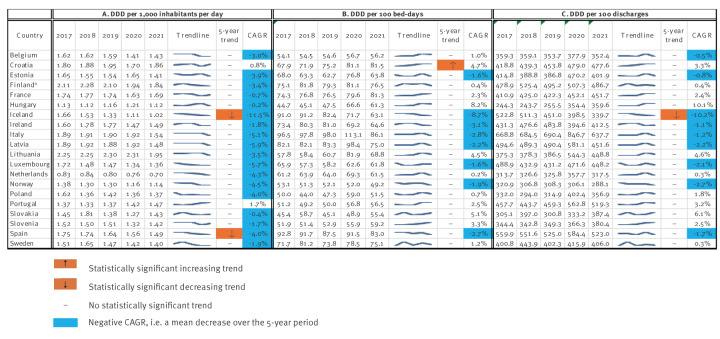
Trends and compound annual growth rate (CAGR) for hospital sector antibiotic consumption rates expressed and ranked as defined daily doses per (A) 1,000 inhabitants per day, (B) 100 bed-days and (C) 100 discharges, 20 EU/EEA countries, 2017–2021

CAGR: compound annual growth rate; DDD: defined daily doses.

^a^ Finland’s hospital consumption data include consumption in remote primary healthcare centers and nursing homes.

The 5-year (2017–2021) trends in hospital sector antibiotic consumption are presented in Figure 2. Only 20 countries had complete data on DDD, inhabitants, bed-days and discharges for all 5 years, and were included in the analysis. Two countries (Iceland and Spain) had a statistically significant decreasing trend when hospital antibiotic consumption was expressed as DDD per 1,000 inhabitants per day; no country had a statistically significant increasing trend; and 18 countries had a negative CAGR value. When hospital antibiotic consumption was expressed as DDD per 100 bed-days, no country had a statistically significant decreasing trend; one country (Croatia) had a statistically significant increasing trend; and eight countries had a negative CAGR value. When hospital antibiotic consumption was expressed as DDD per 100 discharges, one country (Iceland) had a statistically significant decreasing trend; no country had a statistically significant increasing trend; and nine countries had a negative CAGR value.

Consumption of antibacterials for systemic use was obtained using the Anatomical Therapeutic Chemical code J01 as reported to the European Surveillance of Antimicrobial Consumption Network (ESAC-Net) and retrieved from The European Surveillance System (TESSy) database.

The CAGR estimates the mean annual change of antibiotic consumption as a proportion (%) of antibiotic consumption in 2017.

## Discussion

Our study highlights considerable variations in country rankings and trends in hospital sector antibiotic consumption depending on the denominator used. By employing a population-based denominator (‘inhabitants per day’) and two hospital activity-based denominators (‘bed-days’ and ‘discharges’) sourced from Eurostat, we observed variations in country rankings and 5-year trends of hospital antibiotic consumption rates. Presenting antibiotic consumption rates using several metrics can therefore be helpful for understanding hospital antibiotic consumption in the context of each country’s population and hospital activity.

In some instances, findings were seemingly contradictory when comparing antibiotic consumption expressed using different metrics. For example, Bulgaria had the second-highest hospital antibiotic consumption in 2021 when expressed in DDD per 1,000 inhabitants per day, but the second-lowest consumption when expressed in DDD per 100 discharges. These contrasting rankings can be partly explained by Bulgaria’s high hospitalisation rate, reflected in the highest number of discharges per 100,000 inhabitants in 2021 compared with other EU/EEA countries [22].

Moreover, different trendline directions and statistically significant trends over time were observed when using different metrics, as the denominators can independently trend in opposite directions over time. The overall direction of trendlines were opposing for population-based rates vs hospital activity-based rates of antibiotic consumption for about half of the analysed countries. It has been suggested that changes in hospitalisation practices during the COVID-19 pandemic could be associated with such opposing trends [12,13]. A study from Croatia showed that between 2016 and 2020, hospital sector antibiotic consumption expressed as DDD per 1,000 inhabitants per day decreased, whereas an increase was observed for antibiotic consumption expressed as DDD per 100 bed-days [12]. In Hungary, antibiotic consumption during the COVID-19 pandemic increased substantially when using hospital activity-based denominators, while it remained stable when using a population-based denominator [13].

As ‘discharges’ are a component of ‘bed-days’, one might expect similar trends for antibiotic consumption rates calculated with both hospital activity-based denominators. While such consistency was evident in many countries, changes in the national average length-of-stay (ALOS) in hospitals can lead to opposing trends in antibiotic consumption rates by ‘discharges’ and by ‘bed-days’. This occurred in only one country (Belgium). Though trends in hospital consumption were not statistically significant, there was an overall increase in Belgium’s hospital antibiotic consumption when measured as ‘DDDs per 100 bed-days’, but a decrease when measured as ‘DDDs per 100 discharges’. This can be explained by a reduction in Belgium’s ALOS between 2017 and 2021 [18].

While major shifts in demographics over time may affect population-based and hospital activity-based antibiotic consumption rates, our analysis was not designed to assess for the impact of demographic shifts. Analysing data over a longer period of time for countries undergoing dramatic demographic shifts may reveal the influence of increased antibiotic consumption or longer hospitalisations potentially associated with ageing populations. At the same time, countries with an ageing population may shift more healthcare services to outpatient settings. When analysing hospital sector antibiotic consumption to inform antibiotic stewardship actions in hospitals, it is thus recommended to use metrics with denominators specific to the inpatient population in which antibiotic consumption occurred [23]. Additionally, interpretation of trends in hospital antibiotic consumption should include consideration of changes in population or hospital policies over time such as migration waves and intensity of hospital care.

Studies examining hospital sector antibiotic consumption at the national level using different hospital activity denominators are emerging. A retrospective study among acute care hospitals in Catalonia between 2008 and 2016 found divergent trends in antibiotic consumption when using different denominators. Specifically, the study identified a significant increasing trend in DDD per 100 bed-days, primarily attributed to a considerable decrease in ALOS, while antibiotic consumption expressed in DDD per 100 discharges remained stable [24].

Different hospital activity-based denominators offer different insights into the intensity of antibiotic use in hospital settings, enabling the identification of areas for potential antibiotic stewardship interventions. Hospital sector antibiotic consumption expressed as ‘DDD per 100 discharges’ describes the average number of DDD of antibiotics per hospital stay in a country, while ‘DDD per 100 bed-days’ or ‘DDD per 100 patient days’ estimates the proportion of hospitalised patients on a given day who were given one DDD of antibiotics. It is important to acknowledge that these are only estimates, as the actual proportion would be lower if patients received multiple antibiotics or if the dosage exceeded the DDD. A common metric used at the hospital level is ‘days of therapy (DOT) per 1,000 patient days’, can be particularly useful when the administered doses differ from the assigned DDD, although data on DOT are not usually readily available at the national level [25-29].

Presenting national hospital sector antibiotic consumption rates with more than one metric provides a more comprehensive overview of the situation in each country's hospital sector [9]. Our rankings of countries according to hospital antibiotic consumption rates in 2021 underscore the potential caveats of benchmarking national hospital antibiotic consumption rates using just one metric. For two countries with the same annual volume of hospital antibiotic consumption (numerator, number of DDD), the antibiotic consumption rate per 100 bed-days will be lower for the country with the longer ALOS. Presenting hospital consumption rates with both the ‘bed-days’ and ‘discharges’ denominators encourages examination of whether differences in ALOS contribute to differences in consumption rates, or if there might be excess antibiotic prescribing in a country. In countries with shorter ALOS, a high number of DDD per 100 discharges but an average number of DDD per 100 bed-days can be due to an excess number of patients receiving antibiotics. In countries with longer ALOS, a high number of DDD per 100 bed-days but an average number of DDD per 100 discharges can indicate excess antibiotic treatment as well. Such examination of national hospital antibiotic consumption rates can help focus national stewardship measures such as targeting antibiotic prescribing upon hospital admission or ensuring a review of the duration of antibiotic prescriptions.

While using ‘discharges’ as a hospital activity-based denominator removes the influence of ALOS, differences in national hospital discharge data collection and reporting remain. Although Eurostat has standard definitions for ‘bed-days’ and ‘discharges’, discrepancies in the inclusion or exclusion of certain types of patients and counting methodologies exist among the reporting countries [19]. Additionally, when calculating antibiotic consumption rates using ESAC-Net data, it is important to note that the hospitals covered by the antibiotic consumption data reported to ESAC-Net might not correspond with the hospitals covered by Eurostat. Adjustments for such differences were not made in our analyses aimed at evaluating the impact of using different denominators in multi-country antibiotic consumption analyses. Nevertheless, if hospital activity-based denominators are used to report hospital sector antibiotic consumption rates from ESAC-Net data, those denominators should represent the hospital patients covered by the antibiotic consumption data reported to ESAC-Net.

Currently, ESAC-Net presents hospital sector antibiotic consumption expressed in ‘DDD per 1,000 inhabitants per day’, providing a rough estimate of the proportion of the entire population treated in a hospital with a course of antibiotics each day. While this metric helps understand the population-adjusted antibiotic consumption rate in the hospital sector compared with the community sector, the denominator does not align with the specific population under surveillance and ‘at risk’ of receiving antibiotics in hospitals in each country, i.e. hospitalised patients. Consequently, it does not facilitate an accurate understanding of hospital antibiotic consumption in the context of hospital activity, where the frequency of antibiotic exposure is significantly higher than in the community. Therefore, a separate examination of this patient population is necessary when discussing hospital antibiotic consumption.

Our analysis revealed that examining ESAC-Net data with two additional hospital activity-based denominators provides valuable insights for interpreting antibiotic consumption data at the hospital level that were overlooked when relying solely on the population-based denominator. Additionally, we highlighted potential differences in national hospital care practices that might influence antibiotic consumption rates, such as variations in national hospitalisation rates and ALOS. National antibiotic stewardship efforts can therefore identify potential areas for improvement in the hospital sector if hospital activity-based denominators could be used in ESAC-Net analyses.

We identified some key challenges to implementing these hospital denominators at a multi-country level. A major limitation of our analyses was the unavailability of national ‘bed-days’ and ‘discharges’ data aligning with the coverage of hospitals and bed-types for antibiotic consumption data reported to ESAC-Net. Eurostat served as our source for hospital denominators for this analysis, as it is the most comprehensive source of national hospital activity data for EU/EEA countries. However, the national statistics on hospital activity reported to Eurostat do not always align with the hospital coverage of consumption data reported to ESAC-Net. For example, the Eurostat dataset that we used excludes ‘day cases’, while antibiotics consumed for in-hospital surgical procedures without an overnight stay might be reported to ESAC-Net with hospital sector antibiotic consumption data. Inclusion of bed-types such as psychiatric care and long-term care in hospital sector antibiotic consumption data reported to ESAC-Net differs among countries, which not only complicates alignment with hospital activity denominators from Eurostat, but also limits comparability between countries [30].

When antibiotic consumption data are not available for all hospitals, further adjustments are required when using national hospital activity denominators such as those reported to Eurostat. The European Surveillance of Antimicrobial Consumption Network allows reporting of less than 100% national coverage of hospital consumption and the option of reporting data that has been extrapolated to 100% coverage [16]. For our analysis using national hospital activity denominators from Eurostat, we extrapolated national consumption values for countries that did not extrapolate to 100% coverage themselves when reporting to ESAC-Net. Still, we cannot assume that the consumption data used for our extrapolations are representative of national hospital sector antibiotic consumption. It would be more precise to obtain hospital activity data for the subset of hospitals represented in the consumption data reported to ESAC-Net.

A final and important limitation with using Eurostat’s hospital activity data for analyses with ESAC-Net hospital consumption data is timeliness. Countries have up to 2 years after the reference year to report data to Eurostat, a noticeable lag compared with national population data which are finalised each October following the reference year [31]. The European Surveillance of Antimicrobial Consumption Network currently publishes population-based antibiotic consumption rates each November in its Annual Epidemiological Report (AER) using data referring to the previous year. Publication of hospital activity-based antibiotic consumption rates in the AER each November would therefore require countries to report hospital activity denominator data to ESAC-Net separately. Notably, across Europe, antibiotic stewardship programmes at both the hospital and national levels are already examining hospital antibiotic consumption using multiple hospital-specific denominators [5,13,14,32,33]. A feasible methodology for collection of hospital denominator data that is both timely and consistent with the national hospital coverage of ESAC-Net consumption data is currently being explored.

## Conclusion

Despite the methodological challenges, ESAC-Net should strive to report multi-country hospital sector antibiotic consumption rates using hospital activity-based denominators. National surveillance programmes should do the same for a more comprehensive understanding of country-specific hospital antibiotic consumption patterns. Our analysis illustrated how different denominators influence national hospital antibiotic consumption rates, and thus help identify potential areas of excess hospital antibiotic consumption providing inputs for national hospital stewardship interventions. By optimising how we examine and use hospital antibiotic consumption data at the multi-country level, we can optimise efforts to limit the emergence and development of antibiotic resistance in healthcare settings and communities.
